# Expressions of glutathione S-transferase alpha, mu, and pi in brains of medically intractable epileptic patients

**DOI:** 10.1186/1471-2202-9-67

**Published:** 2008-07-18

**Authors:** Wei Shang, Wei-Hong Liu, Xiu-He Zhao, Qin-Jian Sun, Jian-Zhong Bi, Zhao-Fu Chi

**Affiliations:** 1 Department of Neurology, the Second Hospital of Shandong University, Ji'nan, PR China; 2 Department of Neurology, Qilu Hospital of Shandong University, Ji'nan, PR China

## Abstract

**Background:**

Glutathione S-transferases (GSTs) play an important role in metabolizing anti-epileptic drugs (AEDs) in liver. Expressions of GSTs in brain, which may result in poor efficacy of AEDs, have not been well studied. Using clinical cortex specimen from 32 intractable epileptic subjects and 8 non-epileptic controls, the present study investigated the correlation between GSTs and intractable epilepsy.

**Results:**

Three different GST isoforms (α, μ, and π) were detected with immunohistochemistry. GST-α expression was not seen in any cortex specimens. Sixty three percent (63%) of control and 53% of intractible epileptic specimens showed GST-μ immunoreactivity. No significant difference in intensity of GST-μ staining was observed between these two groups. GST-π expression was found in endothelial cells and glial cells/astrocytes. Fifty percent (50%) of the control patients and 66% of the epileptic patients were GST-π positive. The grading of epileptic patients was significantly higher than that of control patients (*p *< 0.01).

**Conclusion:**

High levels of GST-π in endothelial cells and glial cells/astrocyte correlate to medical intractable epilepsy, suggesting that GST-π contributes to resistance to AED treatment.

## Background

Epilepsy is a common neurological disorder, affecting approximately 1 to 2% of the population [1]. The majority of epileptic patients are successfully treated with anti-epileptic drugs (AEDs). Nevertheless, about 20–25% of epileptic patients, as defined as medically intractable epilepsy, fail to respond to AEDs [2].

AEDs can prevent abnormal neuronal firing and seizure spread at seizure focus. The enzymatic activity of Glutathione S-transferases (GSTs) in liver plays an essential role in metabolizing and clearing AEDs [3-5]. GSTs are a group of phase II enzymes of defense that catalyze the conjugation of reduced glutathione to a wide range of electrophiles [6]. There are eight isoforms of soluble GST (α, μ, π, θ, ω, ζ, σ, and κ) and at least three membrane bound GST isoforms (MGST1, MGST2 and MGST3) [7,8]. GSTs are widely expressed in almost every tissue, while some isoforms show tissue-specific distribution. In mammals, expression of GST-α, μ, and π in CNS was reported [9-12]. In the present study, it is hypothesized that higher levels of GSTs in brain, especially in brain-blood barrier may result in poor intraparenchymal accumulation of AEDs, and lead to medical intractability. Therefore, expression levels of GST-α, μ, and π at cortex from intractable epileptic patients were examined.

## Results

### Epileptic patients and their brain lesions

Thirty two (32) epileptic patients (18 male, and 14 female, aged 8 to 43 years) had been treated for over 2 years with AEDs, such as phenytoin, phenobarbital, carbamazepine, valproate and topiramate. Twenty-seven of them received three drug trials, and the other five patients received four drugs trials. The seizures attacked at least 3 times per month during the last six months. Medical imaging and H&E staining identified structural abnormities in brains of all 32 cases. These structural brain abnormities included 24 cases of hippocampal sclerosis, 5 cases of arteriovenous malformations, 2 cases of old infarct, and 1 case of virus encephalitis. Seizure focuses in cortex were found in 30 cases out of 32 patients. Surgical management successfully removed epileptic associated brain lesions and seizure focuses.

### GST expression in epileptic cortex

**Figure 1:**
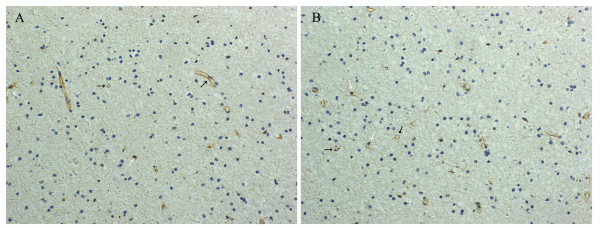
**GST-μ expression in epilepsy group and control group**. Immunohisochemistry was performed to detect GST-μ expression in brain corex sections. GST-μ antibody primarily stains the cytoplasm of capillary endothelial cells(black arrow) and some glial cells(white arrow). (A) parenchymal section from epileptic patient with arteriovenous malformations (magnification ×200). (B) parenchymal section from control patient with arteriovenous malformations(magnification ×200).

**Figure 2:**
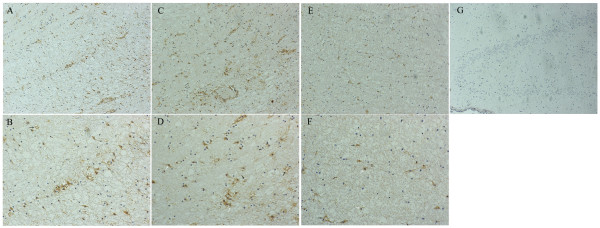
**GST-π expression in epileptic group and control group**. Immunohisochemistry was performed to detect GST-π expression in brain cortex sections with biotin streptavidin-HRP method. Endothelial cells were indicated with black arrow and glial cells/astrocytes were indicated with white arrow. (A, B) GST-π protein expression in temporal subcortex white matter from an epileptic patient with hippocampal sclerosis. Positive GST-π staining was mainly found at the cytoplasm of astrocyte-like cells and capillary endothelial cells (A, magnification ×100; B, magnification ×200). (C,D) GST-π protein expression in temporal subcortex white matter from an epileptic patient with arteriovenous malformations. Immunostaining is in the cytoplasm of astrocyte-like cells and capillary endothelial cells (C, magnification ×100; D, magnification ×200). (E, F) GST-π protein expression in temporal subcortex white matter from a patient of control group. Immunostaining is in capillary endothelial cells and some glial cells (E, magnification ×100; F, magnification ×200). (G) GST-π protein expression in brain from an epileptic patient with hippocampal sclerosis. Immunostaining is not detected in gyrus dentatus of hippocampus. (magnification ×100).

Expression of GSTs in cortical tissues from epilepsy patients were compared with the controls from patients with arteriovenous malformations using immunohistochemistry. GST-α was not detectible in either control specimens or epileptic brain specimens. However, it was detected in positive control specimens (hepatoma tissue). GST-μ immunoreactivity was detected in endothelial cells and glial cells in 63% (5/8) of control patients. The GST-μ staining localized in cytoplasm. Fifty three percent (53%) of epileptic patients showed GST-μ immunoreactivity in endothelial cells and glial cells (Fig. 1). No significant difference in intensity of GST-μ staining was observed between epileptic patients and controls (Table 1, *p *> 0.05). GST-π immunoreactivity was detected in endothelial cells and glial cells with a clear cytoplasmic staining (Fig. 2A and 2B). Fifty percent (50%) of control patients were GST-π positive, while 66% of epileptic patients were GST-π positive. The grading of epileptic patients was significantly higher than that of control patients (Table 2, *p *< 0.01). Because all 8 the controls were patients with arteriovenous malformations, comparison was further made between the subgroup of 5 epileptic patients with arteriovenous malformations and controls. Four epileptic patients showed GST-π immunoreactivity. The intensity of GST-π staining in glial cells was higher in epileptic patients than that in controls (Fig. 2C and 2D), but the grading of epileptic patients was not significantly increased compared to that of control patients (p > 0.05). In controls, GST-π positive glial cells showed less intensity of GST-π staining than endothelial cells (Fig. 2E and 2F). In epileptic group, GST-π positive cells widely distributed in cortex and subcortex white matter. No GST-π-positive neuron or glial cells were found in the hippocampus [Fig. 2G]. GST-π positive cells in epileptic patients showed significant increase in immunoreactivity. The highly GST-π stained glial cells frequently showed morphology of astrocytes (Fig. 2B and 2D).

**Table 1:** Comparison of GST-μ expression between epilepsy group and control group

	Epilepsy case (%)#	Control case (%)
0	15 (46.9)	3 (37.5)
1+	8 (25)	4 (50)
2+	7 (21.9)	1 (12.5)
3+	2 (6.2)	0

# *p *> 0.05, epilepsy vs. control

**Table 2:** Comparison of GST-π expression between epilepsy group and control group

	Epilepsy case (%)*	Control case (%)
0	11 (34.4)	4 (50)
1+	2 (6.2)	4 (50)
2+	8 (25)	0
3+	11 (34.4)	0

* *p *< 0.01, epilepsy vs. control

## Discussion

Because of the lack of response to antiepileptic drugs (AEDs), medical intractability is a crucial clinical problem in human epilepsy therapy. One of the most important mechanisms of intractability is the poor penetration of AEDs into brain. It has been shown that GST enzymatic activity in liver plays an essential role in clearing AEDs from blood. Expression of GSTs in brain tissue, especially at the brain-blood barrier may provide critical role in preventing AEDs from reaching seizure focus. Therefore, the present study investigated expression patterns of GSTs in brains of intractable epileptic patients.

Epilepsy is characterized by recurrent unprovoked seizures. It starts from seizure focus, where a sudden, excessive discharge of cortical neurons appear. The majority of seizure focuses are found at cortex. In the present study, 30 out of 32 intractable epileptic patients have cortical seizure focuses. To examine the role of GSTs in development of intractability, expression of GST-α, μ, and π at seizure focuses were investigated with immunohistochemistry, and were compared with control normal cortex. GST-α mainly exists in the liver, and is responsible for detoxication. In the present study, we found GST-α is not present in brain. Though cortex expresses GST-μ, it does not correlate with intractability. GST-π is expressed by cerebral endothelial cells and glial cells. Expression of GST-π was significantly higher in epileptic brain specimens than in controls. Elevated GST-π in glial cells in seizure focus was more evident. Those GST-π positive glial cells showed a typical morphology of astrocytes. Noticeably, GST-π positive astrocytes widely spread at seizure focuses. As they are the major components of the brain-blood barrier, endothelial cells and astrocytes directly affect the intraparenchymal accumulation of AEDs. Therefore, elevated GST-π in those cells may greatly reduce the efficacy of AEDs. Upregulation of GST-π may be one of the key components that contribute to intractability.

Epilepsy develops intractability with the AED treatment. It takes various amount of time for epilepsy to become intractable. It is still unknown how epilepsy acquires intractability. The correlation between GST-π and intractability may suggest that epilepsy acquires the intractability by expressing high level of GST-π in the brain. Thus, GST-π-inducing ability could be associated with the efficacy of AEDs. The better AEDs could be that have low GST-π-inducing ability. Moreover, targeting GST-π may also improve treatment outcomes in individuals with intractable epilepsy. Although some GST isoforms at liver, testis, and brain tissues were reported to be induced by AEDs in animal studies [13-15], the induction of GST-π by AEDs remains to be studied.

## Conclusion

High levels of GST-π in endothelial cells and glial cells/astrocyte correlate to medical intractable epilepsy, suggesting that GST-π contributes to resistance to AED treatment.

## Methods

### Diagnosis and surgical management of medically intractable epilepsy

All patients took standard neuropsychological tests, ictal and interictal electroencephalogram (EEG) recording. Cranial magnetic resonance imaging (MRI) was performed to identify primary epileptic lesions. When necessary, ictal and interictal single-photon emission computed tomography (SPECT) or positron emission computed tomography (PET) was performed for the same purpose. During the operation, electrocorticogram was performed to localize seizure focuses. Epilepsy-associated lesions and seizure focus were removed by surgery. All procedures were approved and supervised by the Second Affiliated Hospital Ethical Committee of Shandong University, and were performed with patients acknowledgement and written consent.

### Tissue collection and preparation

Brain specimens were obtained from 32 intractable patients and eight control patients with arteriovenous malformations that underwent surgical treatment. Brain specimens were fixed with formalin and paraffin-embedded. The specimens were sectioned at 5 μm.

### Histology and immunohistochemistry

The pathological alterations of primary epileptic lesions were investigated with hematoxylin and eosin (H&E) staining and microscopy. Expression of GSTs was detected using immunohistochemistry with HistoMark Biotin Streptavidin-HRP Systems (Gaithersburg, Maryland, USA). Antibodies against GST-α, μ, and π were obtained from Lab Vision Corporation, Fremont, California, USA. DAB was used as a chromogen. Nuclei were visualized by hematoxylin counterstaining. Positive and negative controls were included in each experiment. All sections were processed simultaneously to ensure standard staining conditions. All slides were examined by two pathologists and two neurologists separately and together. To quantify, the percentages of positively stained cells were calculated in 5 random fields of each section of cortex. The percentages were graded on a scale of 0–3+: 0, no cells stained; 1+, ≤10% of cells stained; 2+, 10–25% of cells stained; 3+, >25% of cells stained.

### Statistics

All data were analyzed with Ridit test. *P *< 0.05 was considered significant.

## Authors' contributions

WS, J–ZB, and Z–FC contributed to conception and study design, WS, W–HL, X–HZ, and Q–JS conducted experiments and data analyses; WS and Z–FC wrote the paper.
